# An Integrated Omics Approach Uncovers the Novel Effector *Ecp20-2* Required for Full Virulence of *Cladosporium fulvum* on Tomato

**DOI:** 10.3389/fmicb.2022.919809

**Published:** 2022-07-05

**Authors:** Mansoor Karimi-Jashni, Kazuya Maeda, Farzaneh Yazdanpanah, Pierre J. G. M. de Wit, Yuichiro Iida

**Affiliations:** ^1^Department of Plant Pathology, Tarbiat Modares University, Tehran, Iran; ^2^Laboratory of Plant Pathology, Setsunan University, Hirakata, Japan; ^3^Department of Cell and Molecular Biology, Shahid Beheshti University, Tehran, Iran; ^4^Laboratory of Phytopathology, Wageningen University, Wageningen, Netherlands

**Keywords:** tomato pathogen, homology modeling, qPCR, RNA-sequencing, fungal effector

## Abstract

The fungus *Cladosporium fulvum* causes the leaf mould in tomatoes. During the colonization of the host, it secretes plenty of effector proteins into the plant apoplast to suppress the plant’s immune system. Here, we characterized and functionally analyzed the *Ecp20-2* gene of *C. fulvum* using combined omics approaches. RNA-sequencing of susceptible tomato plants inoculated with *C. fulvum* race 0WU showed strongly induced expression of the *Ecp20-2* gene. Strong upregulation of expression of the *Ecp20-2* gene was confirmed by qPCR, and levels were comparable to those of other known effectors of *C. fulvum*. The *Ecp20-2* gene encodes a small secreted protein of 149 amino acids with a predicted signal peptide of 17 amino acids. Mass spectrometry of apoplastic fluids from infected tomato leaves revealed the presence of several peptides originating from the Ecp20-2 protein, indicating that the protein is secreted and likely functions in the apoplast. In the genome of *C. fulvum*, *Ecp20-2* is surrounded by various repetitive elements, but no allelic variation was detected in the coding region of *Ecp20-2* among 120 *C. fulvum* isolates collected in Japan. Δ*ecp20-2* deletion mutants of strain 0WU of *C. fulvum* showed decreased virulence, supporting that Ecp20-2 is an effector required for full virulence of the fungus. Virulence assays confirmed a significant reduction of fungal biomass in plants inoculated with Δ*ecp20-2* mutants compared to those inoculated with wild-type, Δ*ecp20-2*-complemented mutants, and ectopic transformants. Sequence similarity analysis showed the presence of *Ecp20-2* homologs in the genomes of several Dothideomycete fungi. The Ecp20-2 protein shows the best 3D homology with the PevD1 effector of *Verticillium dahliae*, which interacts with and inhibits the activity of the pathogenesis-related protein PR5, which is involved in the immunity of several host plants.

## Introduction

*Cladosporium fulvum* (syn. *Passalora fulva* and *Fulvia fulva*) is a biotrophic fungal pathogen that causes leaf mold in tomatoes (*Solanum lycopersicum*) (Thomma et al., 2005). Under moderate temperatures and high relative humidity, *C. fulvum* causes olive-green spots on the abaxial side of leaves that turn greenish-brown velvet-like when the fungus starts to sporulate. Soon, infected leaves become necrotic and drop-off the plant. *Cladosporium fulvum* enters tomato leaves through stomata and colonizes the apoplastic space surrounding mesophyll cells, where it secretes plenty of effector proteins (de Wit and Spikman, 1982). Most of the known secreted effectors are small proteins with less than 300 amino acids containing four or more cysteine (Cys) residues (de Wit et al., 2012).

The interaction between *C. fulvum* and tomato follows the gene-for-gene relationship. Most tomato cultivars carry resistance genes that encode Cf immune receptors. Effectors are supposed to play a role in virulence, but their perception by these Cf immune receptors often elicits a hypersensitive response (HR), leading to incompatibility and resistance to the pathogen. Thus, an effector becomes an avirulence factor (Avr) in the presence of a matching Cf receptor, which induces host resistance. So far, six tomato *Cf* resistance genes (*Cf-2.1/Cf-2.2*, *Cf-4*, *Cf-4E*, *Cf-5*, and *Cf-9*) have been cloned (de Wit et al., 2012). The proteins encoded by these *Cf* genes recognize the matching Avr factors Avr2, Avr4, Avr4E, Avr5, and Avr9 of *C. fulvum*, respectively (Stergiopoulos et al., 2010; Mesarich et al., 2014). Additional potential *Cf-Ecp* resistance genes were discovered in wild accessions of tomato that mediated an HR when infiltrated with the *C. fulvum* effectors Ecp1, Ecp2, Ecp2-1, Ecp3, Ecp4, Ecp5, and Ecp6, respectively. RNA-sequencing (RNAN-Seq.) of *C. fulvum*-infected tomato plants and subsequent bioinformatic analysis uncovered more than 50 *Ecp* genes that are induced *in planta* encoding small effectors, of which nine caused an HR in the wild accession of tomato (Mesarich et al., 2018). These HR-responding tomato accessions are valuable sources for new resistance genes to be introduced in commercial tomato varieties.

Apart from the HR, recognition of effector proteins by Cf immune receptors induces various other defense-related gene families (Jaswal et al., 2020). Tomato chitinases, glucanases, and proteases accumulate abundantly in response to fungal pathogens (Balasubramanian et al., 2012; Cao and Tan, 2019; van der Hoorn and Klemenčič, 2021). Fungal tomato pathogens secrete many different effectors, but the mechanisms of action and targets of only a few are known. These mechanisms can be classified into: (i) offensive inhibitory mechanisms that prevent the activity of host defense enzymes. (ii) Defensive mechanisms that protect fungal cell components against plant enzymes. (iii) An offensive enzymatic mechanism targeting and cleaving host defense proteins. The first two mechanisms have, so far, been reported for two effectors of *C. fulvum*, whereas the last mechanism was reported in other fungal tomato pathogens that secrete proteases that synergistically cleave host defense chitinases (Karimi Jashni et al., 2015b).

So far, 10 effector proteins of *C. fulvum* have been cloned, of which the intrinsic function of only Avr2, Avr4, and Ecp6 has been determined (Mesarich et al., 2018). Avr2 is a cysteine protease inhibitor that inhibits tomato proteases Rcr3 and Pip1, which are involved in host immunity (Rooney et al., 2005; Shabab et al., 2008). Avr4 is a defensive effector that binds to chitin present in fungal cell walls to prevent its cleavage by plant chitinases (van den Burg et al., 2006; van Esse et al., 2007). Ecp6 is a LysM-containing effector that binds and scavenges chitin fragments released from invading hyphae, preventing them from acting as pathogen-associated molecular patterns (PAMPs) (de Jonge et al., 2010; Sánchez-Vallet et al., 2013).

Here, we studied the *Ecp20-2* gene using integrative omics approaches. The *Ecp20-2* gene is one of the three *Ecp20* homologs (*Ecp20-1*, *Ecp20-2*, and *Ecp20-3*) identified by RNA-Seq analysis (Mesarich et al., 2014, 2018). All three genes encode proteins with some homology to Alt A1 allergenic protein. Apart from high expression of the *Ecp20-2* gene *in planta*, mass spectrometry showed that the Ecp20-2 protein is abundantly present in the AF of strain 0WU-infected HCf-0 tomato plants, suggesting it could represent an important virulence factor. Δ*ecp20-2*-deletion mutants showed reduced virulence, while these mutants could be restored to wild type by complementation with the *Ecp20-2* gene. Surprisingly, the *Ecp20-2* gene showed no polymorphism among a collection of 120 strains of *C. fulvum* collected in Japan, while its genomic location is surrounded by repeats like transposable elements. The *Ecp-20-2* gene shows the most homology to the PevD1 protein from *Verticillium dahliae*, which interacts with and protects this fungus against the pathogenesis-related protein PR5.

## Materials and Methods

### Plant and Fungal Material

The fungal strain of *C. fulvum*, race 0 (WU-CBS131901) was used in all expression and virulence studies, and it is present in the stock of our laboratory and is provided by the Laboratory of Phytopathology of Wageningen University. Tomato cultivar Heinz (H)-Cf-0 (susceptible to all races of *C. fulvum*), used for inoculation and expression studies, was provided from the Laboratory of Phytopathology of Wageningen University.

### RNA Sequencing Analysis

The RNA-Seq data from *in vitro*-grown of *C. fulvum* race 0WU on Potato Dextrose Broth (PDB) and from H-Cf-0 (at 6-, 9-, and 12-days post-inoculation (dpi) with *C. fulvum* (strain 0WU) was analyzed as previously described (Mesarich et al., 2018). In short, RNA was sequenced on Illumina platform and using Bowtie version 2-2.1.0 (Langmead and Salzberg, 2012) and TopHat version 2.0.12 (Kim et al., 2013), paired-end reads were mapped to the *C. fulvum* strain 0WU genome (de Wit et al., 2012). Reads were assembled into transcripts, and their abundance was calculated using Cufflinks version 2.0.2 (Trapnell et al., 2010) as FPKM (Fragments Per Kilobase of transcript per Million mapped reads) values. Genes encoding small secreted proteins with a low FPKM value during growth *in vitro* and a high FPKM value during growth *in planta* were considered as candidate effectors and used for further studies.

### Quantitative Real-Time PCR Analysis

Three infected leaflets were sampled from each inoculated plant at 3, 6, 9, 12, 14, and 16 dpi, immediately frozen in liquid nitrogen, and stored at −80°C. Samples were ground to a powder in liquid nitrogen. Total RNA was extracted using the DNase I (Qiagen, Venlo, Netherlands) and the NucleoMag RNA (TakaraBio, Shiga, Japan) according to the manufacturer’s instructions. cDNA was synthesized using the PrimeScript II first-strand cDNA Synthesis Kit (TakaraBio, Shiga, Japan) according to the manufacturer’s instructions. The qPCR analysis was performed using the Thermal Cycler Dice Real-Time System II (TakaraBio, Shiga, Japan) with the KAPA SYBR Fast qPCR Kit (Nippon Genetics, Tokyo, Japan) according to the manufacturer’s instructions. Primers for qPCR were designed using the Primer3plus server as described in Supplementary Table 1. Fungal biomass in tomato leaves was measured based on the amplification of the *C. fulvum* actin gene in 100 ng of total genomic DNA (Mesarich et al., 2014). Tomato ribulose-1, 5-bisphosphate carboxylase/oxygenase (rubisco) gene was used for calibration. Results were analyzed according to the E^–Δ*Ct*^ method (Livak and Schmittgen, 2001) with the average of three biological replicates. Statistical differences in growth between wild-type and Δ*ecp20-2* mutants have been analyzed using the Tukey’s multiple comparison test.

### Mass Spectrometry Analysis

To study the presence and abundance of the Ecp20-2 protein in the AF of *C. fulvum*-infected plants, leaves of 4-week-old H-Cf-0 tomato plants were inoculated with a conidial suspension of a *C. fulvum* strain 0WU (at a concentration of 10^6^/ml). Inoculated plants were incubated in the greenhouse at 22°C with a 16/8-h light/dark regime. Leaflets from visibly infected leaves were collected at 12–15 dpi, and AF was isolated using the previously described method (de Wit and Spikman, 1982; Bolton et al., 2012). Isolated AF was concentrated 10× by freeze-drying and centrifuged at 12,000 × *g* at 4°C for 15 min to remove any precipitate formed during the freeze-drying process. To prepare samples for LC-MS/MS analysis, 100 μl of concentrated AF was loaded on Amicon Ultra-0.5, Ultracel-3 Membrane, 3 kDa (EMD Millipore) for filter-aided trypsin digestion as described previously (Lu et al., 2011). After centrifugation, flow-through containing digested peptides was collected in 2-ml LoBind microcentrifuge tubes (Eppendorf) and stored at −20°C until further analysis. Samples were analyzed using nLC MS/MS with a Proxeon EASY nLC connected to a LTQ-Orbitrap XL (Lu et al., 2011) at the Laboratory of Biochemistry of Wageningen University. MS/MS spectra were searched against a six-frame translation of the repeat-masked *C. fulvum* strain 0WU genome sequence (de Wit et al., 2012) and six-frame translation of the most highly abundant *de novo*–assembled *in vitro* and *in planta* RNA-Seq reads of this fungus (Mesarich et al., 2014), as well as six-frame translation of tomato (*Solanum lycopersicum* cv. H-Cf-0) genome sequence (Sato et al., 2012). Contaminant database was used to determine the sequences of common contaminants: BSA (P02769, bovine serum albumin precursor), trypsin (P00760, bovin), trypsin (P00761, porcine), keratin K22E (P35908, human), keratin K1C9 (P35527, human), keratin K2C1 (P04264, human), and keratin K1CI (P35527, human). Proteins with one or more unique peptides and a false discovery rate of <1% across the sample were considered reliable proteins. Peptides and their abundance belonging to Ecp20-20 were collected for further analysis.

### Fungal Transformation and the Creation of Δ*ecp20-2* Mutants

*Cladosporium fulvum* strain 0WU (CBS131901) was used as a wild-type strain. Genomic DNA of *C. fulvum* was extracted using the NucleoMag Plant (TakaraBio, Shiga, Japan) according to the manufacturer’s instructions. PCR experiments were carried out using the PrimeSTAR GXL DNA Polymerase (TakaraBio, Shiga, Japan) or the TaKaRa Ex Taq (TakaraBio, Shiga, Japan) according to the manufacturer’s instructions. PCR amplicons were purified from agarose gels using the NucleoSpin Gel and PCR Clean-up (TakaraBio, Shiga, Japan). To generate the replacement vector, the upstream (0.5 kb) and downstream regions (0.5 kb) of the *Ecp20-2* gene were amplified from genomic DNA using primer sets Ecp20-2US_F/Ecp20-2US_R and Ecp20-2DS_F/Ecp20-2DS_R, respectively (Supplementary Table 1). Hygromycin-resistance (*hph*) gene and green fluorescent protein (*GFP*) cassettes were amplified from pDONR221_gfp_hyg (Okmen et al., 2013) using primers HYGGFP_ Ecp20-2_F and HYGGFP_ Ecp20_R. These primers contain a 5′ overhang sequence for overlap with sequences at the ends of the linearized plasmid or the *hph/GFP* cassette (Supplementary Table 1). Plasmid pPM43GW (Okmen et al., 2013) was amplified using the primers M13F_reverse and M13R_reverse to linearize and remove the *ccdB* gene that encodes a product lethal to bacterial cells. Four fragments were combined to generate the gene replacement vector pKOEcp20-2 (Supplementary Figure 1A) using the In-Fusion EcoDry Cloning Kit (TakaraBio, Shiga, Japan) and subsequently transformed to electrocompetent *Escherichia coli* DH5α (NIPPON GENE, Tokyo, Japan). The correct orientation of fragments in the final constructs was confirmed by PCR and sequencing. The vector pKOEcp20-2 was introduced into *Agrobacterium tumefaciens* AGL1 cells by electroporation, and *C. fulvum* strain 0WU was transformed through the *A. tumefaciens* strain as previously described (Okmen et al., 2013). Transformants were selected on potato dextrose agar (BD Difco, Franklin Lakes, NJ, United States) supplemented with hygromycin (100 μg/ml) (FUJIFILM Wako Pure Chemical, Osaka, Japan). Transformants were screened by PCR and quantitative real-time PCR (qrtPCR) as shown in Supplementary Figures 1B,C. Strain KO6 (Δ*ecp20-2* mutant) was complemented with a functional *Ecp20-2* gene. A *Ecp20-2* genomic DNA fragment of 2,838 bp (comprising the complete *Ecp20-2* ORF as well as a native promoter and terminator region of 1,874 and 513 bp, respectively) was amplified using primer set proECP20-2_F/termEcp20-2_R. A geneticin-resistance cassette (*gen*) was amplified from pII99 (Namiki et al., 2001) using primers GEN_Ecp20-2_F/GEN_Ecp20-2_R. Two fragments and linearized pPM43GW were combined to generate the gene complement vector pCOEcp20-2 using the In-Fusion EcoDry Cloning Kit (TakaraBio, Shiga, Japan) and subsequently transformed to *E. coli* DH5a (NIPPON GENE). The pCOEcp20-2 was transformed into KO6 as stated above through *A. tumefaciens*, and transformants were screened by PCR and qrtPCR as shown in Supplementary Figures 1B,C. The single insertion event of the gene deletion cassette was confirmed by qPCR using genomic DNA of each transgenic strain extracted as described above. Amplification of the *hph* or *gen* genes hph_qrtPCR_F/hph_qrtPCR_R and GENqrtPCR_F/GENqrtPCR_R, respectively, was used as a measure for the number of insertion events, together with the *C. fulvum* actin gene for normalization as a single copy reference gene, using the 2*^–^*^Δ^ *^Ct^* method (Livak and Schmittgen, 2001). Results are the average of three biological replicates (Supplementary Figure 1C).

### Virulence Assay

*Cladosporium fulvum* strains were cultured on half-strength of potato-dextrose agar (BD, Franklin Lakes, NJ, United States) for 2 weeks in the dark at 22°C, and conidia were collected with distilled water. Conidia were passed through cheesecloth to remove fungal mycelia and centrifuged at 4,000 × *g* for 10 min. Conidia were washed with distilled water, collected by centrifugation twice, and diluted to a final concentration of 1 × 10^6^ conidia/ml. The susceptible tomato *S. lycopersicum* cv. Money Maker Cf-0 was used for the inoculation test. Tomato seedlings at 4 weeks were grown in a climate chamber set to 22°C at approximately 60% relative humidity (RH) with a 16 h light/8 h dark photoperiod. The spore suspension was spray-inoculated onto the abaxial surface of tomato leaves. Three seedlings inoculated with each strain were grown in plastic boxes for 2 days to keep 100% RH in a climate chamber, and boxes were subsequently opened for the remainder of the experiment.

### Analysis of Allelic Variation in Coding Sequence and Repetitive Elements Surrounding the *Ecp20-2* Gene

Allelic variation in the *Ecp20-2* gene was determined for 120 strains of *C. fulvum* (Supplementary Table 2), including a previously reported collection (Iida et al., 2015). Genomic DNA isolation and PCR amplification were described as above. Of note, 618 bp containing the *Ecp20-2* gene was PCR-amplified using primers as described in Supplementary Table 1. PCR amplicons were excised from 0.8% agarose gels and purified from agarose gels using the NucleoSpin Gel and PCR Clean-up (TakaraBio, Shiga, Japan). Sequences were determined using an Applied Biosystems 3730xl DNA analyzer (Thermo Fisher Scientific, Waltham, MA, United States). To evaluate DNA sequences surrounding the *Ecp20-2* gene for the presence of transposable elements (TE), a size of 10 kb sequence upstream and downstream of *Ecp20-2* (scf7180000130934) was obtained from the *C. fulvum* genome at JGI (de Wit et al., 2012). To screen the query sequence against a reference collection of repeats, it was uploaded to RepBas (Kohany et al., 2006) with default settings. Output data revealed the type and class of transposable elements.

### Phylogenetic Analysis

Amino acid sequences of Ecp20-2 were used as query sequences for blastp searches against the Dothodeomycet genome (JGI server). We used the Signalp online server to predict the signal peptide required for secretion (Petersen et al., 2011). For phylogenetic analysis, sequences of each class were aligned using ClustalW, and the curated alignments were used by the Neighbor-Joining method to build phytogenic trees. The evolutionary distances were computed based on the JTT amino acid substitution model and in the units of the number of amino acid substitutions per site. All ambiguous positions were removed for each sequence pair (pairwise deletion option). There were a total of 171 positions in the final dataset. Evolutionary analyzes were conducted in MEGA X (Tamura et al., 2011).

### Structural Homology Modeling

To determine the possible function of Ecp20-2, homology modeling was used to create the 3D structure based on the most similar molecules in the protein databank (PDB). The number of 50 templates for the amino acid sequence of Ecp20-2 was obtained through SWISS-MODEL (Waterhouse et al., 2018). The best template with the highest coverage and QSQE scores was selected and used for building the homology model. The model was visualized through the PyMOL program.

## Results

### RNA-Seq and qPCR Reveal Differential Expression of *Ecp20-2*

**FIGURE 1:**
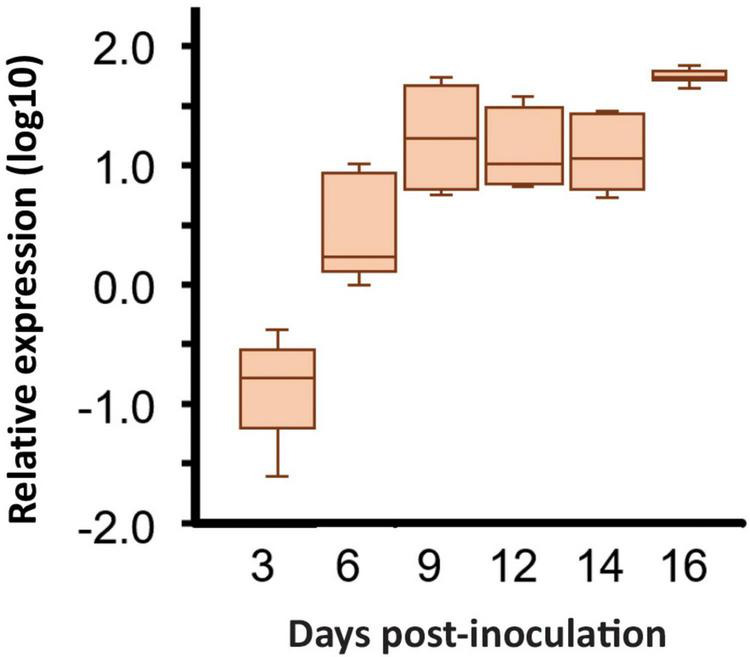
Expression of the *Cladosoprium fulvum Ecp20-2* gene is induced after infection of tomato seedlings. Expression was monitored by a quantitative real-time PCR in tomato plants inoculated with *C. fulvum* strain 0WU at 3-, 6-, 9-, 12-, 14-, and 16-days post-inoculation (dpi). Expression was normalized to that exhibited by the *C. fulvum* actin gene or tomato rubisco gene according to the 2^–ΔCt^ method with the average of three biological replicates. The box plot indicates the median indicated by the horizontal line in the box with the lines indicating the top and bottom 25% of the distribution, whiskers represent the maximum and minimum values, and the outliers are indicated by dots. Statistical differences among strains have been analyzed using the Tukey’s multiple comparison test.

Analysis of RNA-seq data showed that the *Ecp20-2* gene was differentially induced during colonization of susceptible tomato cultivar H-Cf-0. The FPKM values of *Ecp20-2* of the *in vitro*-grown fungus were 56 times lower than those for the fungus during colonization of tomato. The RNA-seq data showed that the expression of *Ecp20-2* followed a similar pattern to known effectors of *C. fulvum* and increased gradually during colonization reaching the highest value at 8 dpi (Supplementary Figure 2). The expression of *Ecp20-2* was quantified using reverse-transcription quantitative real-time PCR (RT-qPCR). The qrtPCR data confirmed the RNA-seq expression patterns showing clear up-regulation of *Ecp20-2* at 9, 12, 14, and 16 dpi. The expression of *Ecp20-2* was equal to the housekeeping gene actin at 3 dpi, but it was strongly induced during infection and peaked at 16 dpi (Figure 1). Expression studies of genes encoding known effectors Avr5 and Avr9 showed a similar induction pattern (Mesarich et al., 2014). These two genes were also lowly expressed in PDB medium and strongly induced during colonization of tomato. This suggests that *Ecp20-2* is most likely involved in fungal virulence rather than fungal physiology and development. When screening the promoter region of *Ecp20-2*, we found five GATA boxes and one TATA box in the 0.6 kb upstream region of this gene (Supplementary Table 2). These boxes were also upstream for the *Avr5* and *Avr9* genes, and are supposed to be binding sites for (TA) GATA-type nitrogen-responsive transcription factors (Pérez-García et al., 2001; Snoeijers et al., 2003).

### Proteomics Supports the Secretion of the Ecp20-2 Protein Into the Apoplast of Tomato During Colonization by *Cladosporium fulvum*

Mass spectrometry analysis of AF obtained from *C. fulvum*-inoculated tomato plants confirmed the presence of Ecp20-2 and suggests that it is secreted into the apoplast during host colonization. The Ecp20-2 protein carries a 17 amino acid signal sequence at its N-terminus suggesting that it is secreted into the apoplast. The detection of five unique peptides belonging to the Ecp20-2 protein by tandem-MS (MS/MS) analysis proved that Ecp20-2 is indeed secreted into the apoplast. The intensity of each peptide in the sample, their charge, and molecular weight are shown in Supplementary Table 3. We used MaxQuant to calculate iBAQ (intensity-based absolute quantification), by dividing protein intensities by the number of theoretically observable tryptic peptides. The iBAQ value is generally highly correlated with the abundance of the protein. The iBAQ for Ecp20-2 was 6.366, and only seven proteins out of 62 identified proteins, originating from *C. fulvum*, had a higher abundance than Ecp20-2.

### Homologous Genes of Ecp20-2 Are Present in a Few Phylogenetically Related Fungi

**FIGURE 2:**
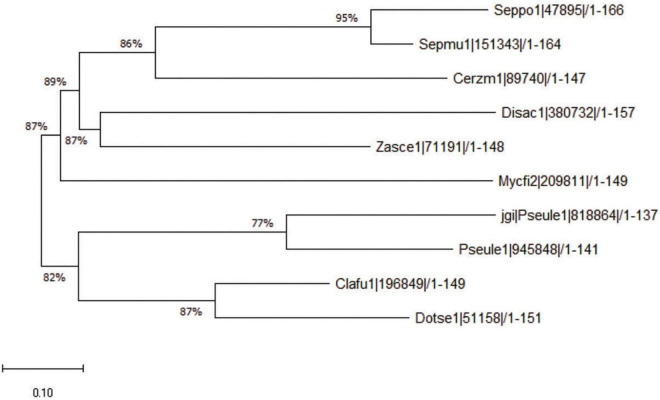
Phylogenetic tree of Ecp20-2 orthologs. The phylogenetic tree resents the similarity of Ecp20-2 amino acid sequence with 9 other orthologs found in the other Dothedeomycetes. Amino acid sequences encoded by orthologous genes were aligned using ClustalW, and the phytogenic tree was constructed by neighbor-joining method in MEGA X. Clafu1, Dotse1, Pseule1, Mycfi2, Zasce1, Disac1, Cerzm1, Sepmu1, and Seppo1 stands for orthologous genes from fungi *Cladosporium fulvum* v1.0, *Dothistroma septosporum* v1.0, *Pseudocercospora ulei* v1.0, *Mycosphaerella fijensis* v2.0, *Zasmidium cellare* v1.0 *Dissoconium aciculare* v1.0, *Cercospora zeae-maydis* v1.0, *Septoria populicola* v1.0, and *Septoria musiva* v1.0, respectively. Numbers show the percentage of similarity between two orthologous genes.

Ecp20-2 contains four cysteine residues expected to form two disulfide bonds in the folded protein. We assessed the amino acids for known Interproscan and Pfam domains; however, sequence similarity revealed that Ecp20-2 is a unique protein with an unknown functional domain. A blast search with the query sequence of *Ecp20-2* at the JGI pipeline found homologous genes in other Dothideomycetes. The phylogenetic tree shows the closets orthologous gene with 87% similarity in the genome of *Dothistroma septosporum*, a close relative of *C. fulvum* (de Wit et al., 2012). Orthologs genes of *Ecp20-2* that are present in the genomes of the other seven Dothideomycetes are shown in Figure 2. All the homologous genes presented here in these fungi were predicted to encode secreted proteins as they all carried a secretion signal sequence.

### *Ecp20-2* Lacks Allelic Variation in a Japanese Collection of 120 *Cladosporium fulvum* Isolates

The *Ecp20-2* gene is located on scaffold scf7180000130934 and contains one exon. To determine potential allelic variation, we sequenced the *Ecp20-2* gene from 120 isolates of a Japanese *C. fulvum* collection. Genomic sequencing of fragments with a size of 500 bp elucidated that *Ecp20-2* is present in all isolates. In addition, we found that the coding sequence of all isolates was identical to that of the wild-type reference isolate WU0 (Supplementary Table 4). One of the isolates was found to contain a thymine insertion (c.-23insT) 23 bp before the start codon (Supplementary Table 4). To analyze whether the *Ecp20-2* gene is surrounded by transposable elements (TE), a 10-kb sequence upstream and downstream of *Ecp20-2* (scf7180000130934-1547) was analyzed using RepBas, a repeat masker tool with the ability to screen query sequences against a reference collection of repeats that reports their classes as well (Kohany et al., 2006). Results revealed two families of long terminal repeat (LTR) and non-LTR (or NILS) transposons surrounding *Ecp20-2* (Supplementary Figure 3). Seven LTR members belong to the Copia, Gypsy, BEL, and Juno2 classes, and three non-LTR and DNA transposons, including Harbinger and Mad, were predicted by RepBas.

### Ecp20-2 Is Required for the Full Virulence of *Cladosporium fulvum*

**FIGURE 3:**
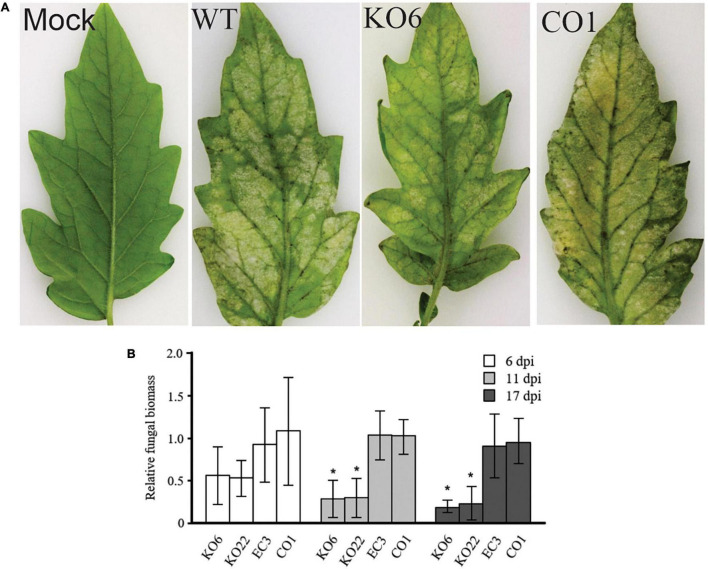
Virulence assay of Δ*Ecp20-2* mutant strains of *Cladosporium fulvum* on tomato. **(A)** A representative picture showing MM-Cf-0 tomato plants 21 days after inoculation with wild-type strain 0WU; KO6, lacks a functional *Ecp20-2* gene; EC3, ectopic transformant; CO1, KO6 complemented with the functional *Ecp20-2* gene. A total of 15–20 leaves of three independent plants (three replications) were inoculated with conidia of *C. fulvum*. **(B)** Fungal biomass measurement by quantitative real-time PCR at 6-, 11-, and 17-days post-inoculation. The degree of fungal colonization was determined by the transcript levels of the constitutively expressed *C. fulvum* actin gene relative to the constitutively expressed tomato ribulose-1, 5-bisphosphate carboxylase/oxygenase (rubisco) gene in 100 ng of total genomic DNA from each sample. For each time point, fungal biomass was plotted relative to that of wild-type, which was set to 1.0. Bars represent mean values and standard deviation. The value of the column labeled with an asterisk differed significantly compared with those of the wild-type, EC3, and CO1 transformants (*P* < 0.05, Tukey’s multiple comparison test).

As the *Ecp20-2* gene is strongly induced during colonization of tomato by *C. fulvum*, and the encoded protein is abundantly present in the apoplast of infected leaves, we reasoned that the protein might be a virulence factor. Therefore, we created three Δ*ecp20-2* mutants of strain 0WU by homologous recombination. These Δ*ecp20-2* mutants, as well as an ectopic transformant and three Δ*ecp20-2* mutants complemented with a single functional copy of the *Ecp20-2* gene, were inoculated onto MM-Cf-0 seedlings to assay their virulence (three biological replications). Representative phenotypic data (Figure 3A) showed full velvet and sporulation at the lower surface of leaves inoculated with the wild type strain 0WU, ectopic transformant, and complemented mutants. However, leaves inoculated with the Δ*ecp20-2* mutants showed reduced signs and symptoms. We assessed the fungal biomass in inoculated plants using qPCR at 6, 11, and 17 dpi. Results confirmed a significant reduction of fungal biomass in the Δ*ecp20-2* mutants compared to wild type, complemented, and ectopic strains (Figure 3B). Deletion of the Ecp20-2 gene delayed colonization of the plant until 21 dpi (Supplementary Figure 4).

### Homology Modeling Revealed a Structural Similarity of Ecp20-2 to the PevD1 Effector of *Verticillium dahliae*

**FIGURE 4:**
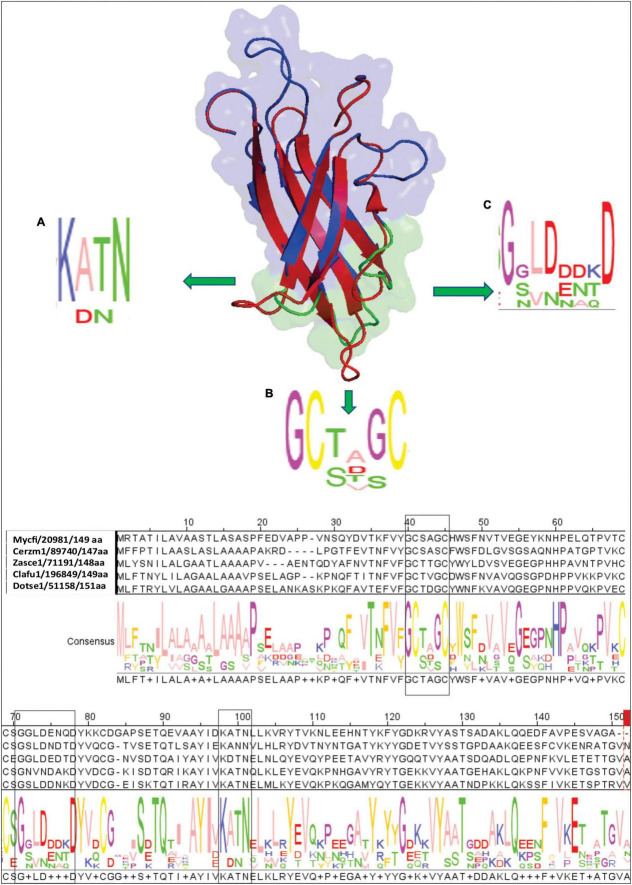
Homology structural model of Ecp20-2. To generate the three-dimensional model, the amino acid sequence without signal peptide of Ecp20-2 was submitted to SWISS-MODEL online server. The best model was built based on the template of effector PevD1 with SMTL ID 5xmz.1 and illustrated by PyMol. The upper side shows a Cartoon view of a superimposed model of Ecp20-2 (in blue) with the PevD1 (in red) structure. Panels **(A–C)** show three loops of Ecp20-2 (in green) expected to be involved in interaction with other molecules. Consensus regions and residues conserved among homologs in other fungi were shown in panels **(A–C)**. The lower part is the alignment of the Ecp20-2 sequence with boxed consensus regions.

The majority of effectors from *C. fulvum* and other plant fungal pathogens do not share conserved signatures in their sequence with those of known effectors. Sequence similarity searches showed that Ecp20-2 lacks conserved signatures of functional domains. We reasoned that a three-dimensional (3D) homology model for Ecp20-2 may help to predict its function. Therefore, we set out to obtain the most identical 3D structures available at the Protein Database (PDB). Assessment using the amino acid sequence of Ecp20-2 at the SWISS-MODEL server led to the discovery of the *V. dahliae* effector PevD1 with SMTL ID 5xmz.1, as the best template with the highest score and stability. Based on the solved 3D structure of PevD1, we built a model for Ecp20-2 and visualized it in PayMol. The superimposed cartoon structure of Ecp20-2 (in blue) and PevD1 (in red) suggests that the six beta-sheets of Ecp20-2 overlay well with those of PevD1 (Figure 4). Like Ecp20-2, PevD1 contains four cysteine residues proposed to be involved in two disulfide bonds. The model determined for PevD1-GhPR5 shows that the N-terminus and C-terminus were folded on the same side of protein and the opposite side of the protein contains the interaction site with the host GhPR5 (Zhang et al., 2019). We evaluated the topology and amino acid sequence of Ecp20-2 at the interaction site predicted for PevD1-GhPR5. Ecp20-2 contains three loops with residues that are mainly conserved among homologs of Ecp20-2 (shown as A–C consensus regions in Figure 4). Both PevD1 and Ecp20-2 are small molecules with a molecular weight of 16 and 14 kDa, respectively. PevD1 is an extracellular effector protein and is involved in the virulence of *V. dahliae* on cotton. It is suggested that PevD1 contributes to virulence through interaction with GhPR5 to protect the fungus against its antifungal activity (Zhang et al., 2019). Recent studies showed the recognition of PevD1-mediated by host signaling molecule NRP regulates the sesquiterpenoid phytoalexins biosynthesis pathway and induces host defense in *Nicotiana benthamiana* (Liang et al., 2021). PevD1 also manipulates the host ORE1-ACS6 cascade, promoting ethylene biosynthesis and leaf senescence in plants (Zhang et al., 2021).

## Discussion

Fungal pathogens secrete plenty of effectors to deregulate the host immune system and to promote host colonization. *Cladosporium fulvum* is a biotrophic fungus that causes leaf mold of tomatoes and secretes various effector proteins in the apoplast. Transcriptome sequencing analysis showed that the *Ecp20-2* gene was lowly expressed during growth on synthetic medium but strongly expressed during the infection of susceptible tomato. We performed qrtPCR to quantify the expression pattern of *Ecp20-2*. Compared with previous studies (Mesarich et al., 2014), *Ecp20-2* follows an expression pattern similar to that of *Avr5* and *Avr9* during the colonization of tomato, suggesting they are required for full virulence and development of *C. fulvum*. The similarity of their expression patterns may suggest that they are under the control of the same transcription factor (TF) or are regulated by different TFs responsive to the same growth conditions. The promoter region of *Avr5*, *Avr9*, and *Ecp20*-2 contain GA(TATA) boxes, previously found as binding sites for nitrogen-responsive transcription factors, including nitrogen response factor 1 (Nrf1) of *C. fulvum* (Pérez-García et al., 2001; Snoeijers et al., 2003). The protein encoded by the *Ecp20-2* gene contains a predicted 17 amino acid signal peptide for secretion. Indeed, LC-MS/MS analysis showed that the Ecp20-2 protein is abundantly present in the AF of infected tomato leaves, which is in agreement with its high level of expression. It also suggests that the secreted Ecp20-2 protein is fairly stable in the harsh environment of the apoplastic space, which might be due to two disulfide bonds potentially present in the protein. The *Ecp20-2* is induced during all phases of tomato colonization between 6 and 16 dpi, suggesting it plays an important role in virulence and apoplastic colonization. Indeed, the Δ*ecp20-2* mutants showed reduced virulence and could be restored to full virulence by complementation with the wild-type *Ecp20-2* gene.

Almost all effectors of *C. fulvum* described so far do not contain functional domains in their protein sequence, except Avr4 and Ecp6, which contain chitin-binding and LysM domains, respectively (de Jonge et al., 2010; de Wit et al., 2012; Kohler et al., 2016). A blast search in the public databases showed that the Ecp20-2 protein shows the most homology with the secreted PevD1 (for protein elicitor from *V. dahliae*) protein of *V. dahliae.* To predict the specific function of Ecp20-2, we applied homology modeling based on the solved structure of the most similar protein present at the PDB database (Waterhouse et al., 2018). Indeed, the best 3D model of Ecp20-2 was built based on the template structures of the Verticillium effector PevD1. PevD1 is an extracellular effector protein of and mainly localizes to the plant plasma membrane but is also found in the cytoplasm, where it interacts with a plant signaling molecule, NRP, leading to a hypersensitive response (Zhou et al., 2017). PevD1 induces a necrotic response in several plants, including cotton and tobacco, and mediates the expression of PR and senescence genes by interaction with various transcription factors. PevD1 has been shown to interact with the senescence-associated NAC transcription factor ORE1 inducing ethylene production and plant senescence (Zhang et al., 2021). PevD1 has also reported to interact with the PR5 protein of cotton, thereby suppressing its antifungal activity (Zhang et al., 2019). So far, tomato receptors that recognize secreted effectors of *C. fulvum* are located in the plasma membrane and are known as receptor-like proteins (RLPs) (Wulff et al., 2009; Stergiopoulos et al., 2010). In 2018, we have cloned 41 apoplastic *in planta*-induced genes including *Ecp20-2* to screen wild tomato accessions to identify new *Cf-Ecp* genes (Mesarich et al., 2018). PVX (potato virus X)-based transient expression showed that nine of these *Ecp* genes induced an HR in at least one of 25 screened tomato accessions (Mesarich et al., 2018). Ecp20-2 did induce an HR in none of the accessions; however, screening other tomato accessions may result in finding a corresponding *Cf-Ecp20-2* gene. In the future, we will also delete paralogs *Ecp20-1* and *Ecp20-3* to see whether they might also have effects on the virulence of *C. fulvum*.

The genome of *C. fulvum* contains a massive increase in repetitive elements, comprising repeat families. Long terminal repeats (LTR) and long interspersed elements (LINEs) are DNA transposon elements. LTR retrotransposons and LINE transposons comprise 90% and 4.7% of the overall repetitive elements in *C. fulvum*, respectively. Both Copia and Gypsy LTR retroelements are expanded in *C. fulvum* (de Wit et al., 2012). Most of the known *C. fulvum* effector genes are located in the repeat-rich regions of the genome and might have been instrumental in their birth during evolution. Indeed, in the 10 kb surrounding the *Ecp20-2* gene, we detected 10 repetitive elements including seven LTRs and three non-LTR and DNA elements. A mechanism called repeat-induced point mutation (RIP) was shown to prevent repeat expansion through G:C to A:T mutation transition in haploid nuclei during the sexual stage (Freitag et al., 2002). Therefore, in Dothideomycete fungi, in which the sexual stage is rare or absent, RIP spillage may not properly prevent the expansion of repetitive elements. RIP occurs in regions adjacent to repetitive elements where effector-encoding genes are located. RIP mutations have occurred in known effectors of *Leptosphaeria maculans*, enabling the fungus to escape host receptor-mediated resistance (Rouxel et al., 2011). We studied the allelic variation of the *Ecp20-2* gene in 120 *C. fulvum* isolates collected in Japan but surprisingly did not find any variation in the gene. The *C. fulvum* genome contains 25.9-Mb RIP regions, mainly occurring on large repeated sequences (de Wit et al., 2012). Homologs in other fungi were sought using tblastn/blastp with the sequences of *Ecp20-2* as a query. The best ortholog with the highest score is present in closely related fungi *Dothistroma septosporum*, *Septoria musiva*, and *Septoria populicola.* Other known effectors of *C. fulvum Avr4*, *Ecp2*, and *Ecp6* also have orthologs in other Dothideomycete fungi. Tomato plants carrying the *Cf-Ecp2-1* and *Cf-4* resistance genes recognize the orthologous effectors DsEcp2-1 and DsAvr4, respectively (de Wit et al., 2012). Also, PfAvr4 of the tomato pathogen *Pseudocercospora fuligena* induces Cf-4-mediated resistance (Kohler et al., 2016). Some orthologs of Ecp20-2 may also be recognized by Cf immune receptors in wild relatives of tomato. In summary, data presented in this and previous studies show that *C. fulvum* secretes different effectors to promote virulence on tomatoes. Here, we characterized *Ecp20-2* using the combined genomics, transcriptomics, proteomics, and bioinformatics approach. We showed that the *Ecp20-2* gene is highly expressed *in planta* and encodes a protein that is secreted and accumulated in the apoplast of tomato, where it is required for full virulence of *C. fulvum*. The *Ecp20-2* gene is surrounded by repetitive elements but did not show allelic variation among 120 *C. fulvum* isolates. Homology modeling suggests that Ecp20-2 is involved in host immunity. During co-evolution, *C. fulvum* has developed many effector proteins to target the inhibitory or hydrolytic activity of host proteins (Karimi Jashni et al., 2015a; De Wit, 2016). Tomato apoplast is a harsh battlefield and contains various PR proteins with antimicrobial roles (Karimi Jashni et al., 2015b). Some of these proteins may function as gardees or decoys in the recognition of fungal effectors (van der Hoorn and Kamoun, 2008). As it has a role in virulence, we expect that Ecp20-2 presumably has apoplastic targets in the host plant. Feasible methods like molecular docking, yeast two-hybrid screening, and Co-IP experiments may help to determine the function and interactors of the Ecp20-2 effector in future studies.

## Data Availability Statement

The datasets presented in this study can be found in online repositories. The names of the repository/repositories and accession number(s) can be found below: https://www.ncbi.nlm.nih.gov/genbank/, ON088852 https://www.ncbi.nlm.nih.gov/, SAMN02642556 to SAMN02642561.

## Author Contributions

MK-J conceived the project. MK-J, KM, FY, PdW, and YI carried out the experimental work, and performed data analysis and interpretation. MK-J, FY, PdW, and YI wrote and edited the manuscript. All methods were carried out in accordance with relevant guidelines in the “Materials and Methods” section. All authors read and approved the submitted version.

## Conflict of Interest

The authors declare that the research was conducted in the absence of any commercial or financial relationships that could be construed as a potential conflict of interest.

## Publisher’s Note

All claims expressed in this article are solely those of the authors and do not necessarily represent those of their affiliated organizations, or those of the publisher, the editors and the reviewers. Any product that may be evaluated in this article, or claim that may be made by its manufacturer, is not guaranteed or endorsed by the publisher.
