# Supplementary material for: An Integrated Omics Approach Uncovers the Novel Effector Ecp20-2 Required for Full Virulence of Cladosporium fulvum on Tomato
Source: Front Microbiol. 2022 Jul 5;13:919809. doi: 10.3389/fmicb.2022.919809 (PMC9294515; doi:10.3389/fmicb.2022.919809)
Supplement: Supplementary file 1 [file Data_Sheet_1.docx]

**Supplementary Material**

**Table S1**. Primers used in this study.

| Name | Sequence (5′→3′) ^a^ | Purpose |
| --- | --- | --- |
| ECP20-2US_F | aaacagctatgaccacgggtgttcaggacgaattg | Amplification of the upstream of  *ECP20-2* gene for pKO *ECP20-2* |
| ECP20-2US_R | ccgggaaccagttaagattgctggggtgcggcgaa |  |
| ECP20-2DS_F | gttcaaacatttggcccagagaatcaaagcctaca | Amplification of the downstream of ECP20-2 gene for *pKO ECP20-2* |
| ECP20-2DS_R | acgacggccagtgaagacgtcaggatgttcagttg |  |
| HYGGFP_ ECP20-2_F | ttaactggttcccggtcggcatcta | Amplification of *hph*/*gfp* cassettes for *pKO ECP20-2* |
| HYGGFP_ ECP20-2_R | gccaaatgtttgaacgatctgcagc |  |
| M13F_reverse | ttcactggccgtcgttttacaacg | Linearization of pPM43GW |
| M13R_reverse | tggtcatagctgtttcctgtgtg |  |
| US_F1 | ttacttcagagcagctccttgctc | Screening of *pKO ECP20-2* transformants (Supplemental figure S1) |
| US_F2 | gctgtatacaggctaggcagaca |  |
| US_R | tacaagccagcaacaccatgtgtc |  |
| DS_F | acttgctgcagctgtaccat |  |
| DS_R | gacttgtcccacagctatgcat |  |
| HPH_R | actcgtccgagggcaaaggaata |  |
| GFP_F | ggcatggacgagctgtacaagta |  |
| pro ECP20-2_F | aaacagctatgaccattacttcagagcagctcctt | Amplification of  *ECP20-2* gene including promoter and terminator for Ecp20-2 |
| Term ECP20-2_R | ttcacggcataggaggtttcgacttgtcccacagc |  |
| GEN_ ECP20-2_F | gctgtgggacaagtcgaaacctcctatgccgtgaa | Amplification of geneticin resistance cassette for *pCO ECP20-2* |
| GEN_ ECP20-2_R | acgacggccagtgaatacctgtgcattctgggtaa |  |
| Cf-actin_RT-qrtPCR_F | ggcaccaatcaacccaaag | qrtPCR (Mesarich et al., 2014) |
| Cf-actin_RT-qrtPCR_R | tacgaccagaagcgtacag |  |
| Sl-rubisco_qrtPCR_F | gaacagtttctcactgttgac |  |
| Sl-rubisco_qrtPCR_R | cgtgagaaccataagtcacc |  |
| Ecp20-2_qPCR_F1 | ccgaaaccaaaccaattcac |  |
| Ecp20-2_qPCR_R1 | acgttaccggagcatttcac |  |
| hph_qrtPCR_F | ataggtcaggctctcgctga |  |
| hph_qrtPCR_R | gatgtaggagggcgtggat |  |
| GENqrtPCR_F | atgactgggcacaacagaca | qrtPCR (Jashni et al., 2015) |
| GENqrtPCR_R | agtgacaacgtcgagcacag |  |

^a^ An overhang sequence in each primer for overlap with sequences at the ends of the linearized plasmid, *hph/gfp* cassettes, or genes is underlined.

**Table S2.** GATA/TATA boxes in the promoter region of  *Ecp20-2.* The size of 0.6kb from the promoter region is highlighted in grey and the coding region is highlighted in red.

CAAAATCCCATGCCGAAGCGTCAACACCAACTTCTCGGCAACACAACGTGTTTTTACGC**GATA**TGAGTATGCACAATATGGAAACA**GATA**GCTGCGAACACATTGGGAAAACAGTCAAGTT**GATA**TGAGCAATATTGGTGGTGTTGAT**GATA**AAAAGTCACTGGGGTGCGGCGATTGCTGGGGTGCGGCGAAGCTTGTGGCCTCGTAATCGACTCCAAGTTATCCCTTTGCATTTCCGTTGACAAACCTATTCACCCTGCTTACAGGGCTTTAAGCTGGATTACGTTCACAAAATTGCCTGTGCAAGCCATGCCGCGACAGACCGAACCAGTCGTGCCAGTCAGACTCTGTCGTTGTCAAGCAACGTTCAAAACTTAGACGGGGCCTCCGCACTGACTTGGACCGACAAAAGGTGCGAAGGGATGATTCTAGGCCCACAAGCCCGAAGCCGCTCGTATTTGCAAC**GATA**CCTTGCGTTCGCTATTCATTGCGAATTAACTCAGTAGGCATACAGCGTCTTCATGCAGCCGAGTAAAATGCGCTTTTAGCTGCCGCCAGATCGATGTTGGCTTATTGGCTC**TATA**CCTGGATTTTGCCAGGATGATCGGTCCGCCGGGATGTAAAATGTATTTCGGCTGCTCGAAGACGCTGTAGATAAAATGGAGCGTTCTGCTCCGCCCAAGACCGCAGCGCCCACATCCTCTTTCTCGAACCAGTCATTAAAACAACAGGACATCGATCAACAGCTCAACTACCAGCTTTCGGCCTCATCACAATGCTCTTCACCAACTATCTCATCCTCGCCGGCGCAGCACTTGCTGCAGCTGTACCATCGGAGCTAGCCGGGCCGAAACCAAACCAATTCACAATTACGAACTTCGTCTTTGGATGCACAGTTGGCTGTGATTGGAGCTTCAACGTTGCTGTGCAAGGATCTGGGCCCGACCATCCGCCTGTCAAGAAGCCAGTGAAATGCTCCGGTAACGTGAATGACGCCAAAGACTACGTCGACTGCGGCAAAATCAGTGATACCCAGAGAATCAAAGCCTACATCGTCAAGGCCACGAATCAGCTCAAGCTTGAGTACGAGGTGCAGAAGCCGAATCATGGTGCCGTGTACCGATACACGGGCGAGAAGAAGGTATATGCCGCCACAGGTGAACACGCGAAGTTACAGAAGCCCAACTTCGTCGTGAAGGAGACCGGCTCGACAGGTGTCGCCTAG

**Table S3.** Peptides secreted from Ecp20-2 were identified by MS/MS analysis.


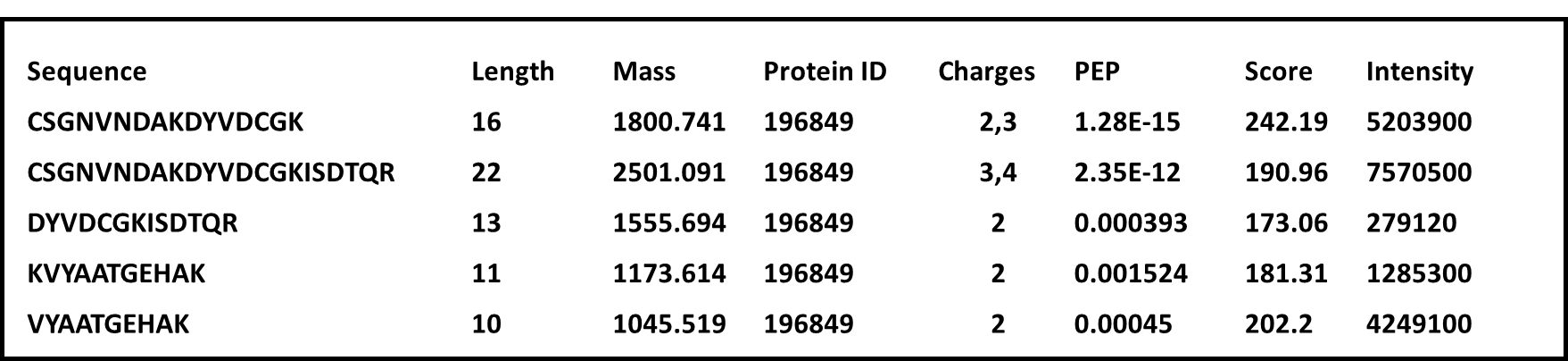


**Table S4**. Allelic variation of *Ecp20-2* in *Cladosporium fulvum* isolates

|  | Isolated date | MAFF number (strain stock center) | Race | MAT1-1orMAT1-2 | Ecp20-2 ^a^ |  |
| --- | --- | --- | --- | --- | --- | --- |
| CF248 | 2008.5.26 | 242503 | 4 | MAT1-2 | wild-type |  |
| CF256 | 2008.5.22 | 242508 | 4.11 | MAT1-2 | wild-type |  |
| CF258 | 2008.5.22 | 726785 | 4.11 | - | wild-type |  |
| CF260 | 2008.5.20 | 726786 | 4.11 | MAT1-2 | wild-type |  |
| CF262 | 2008.5.22 | 726787 | 4.11 | MAT1-2 | wild-type |  |
| CF265 | 2008.5.28 | 242511 | 4.11 | MAT1-2 | wild-type |  |
| CF289 | 2008.6.5 | 726788 | 4.11 | MAT1-2 | wild-type |  |
| CF298 | 2008.6 | 242525 | 4.11 | MAT1-2 | wild-type |  |
| CF300 | 2008.6 | 242527 | 0 | MAT1-2 | wild-type |  |
| CF301 | 2008.6.27 | 242528 | 0 | MAT1-1 | wild-type |  |
| AP26 | 2007 | 726790 | 2 | MAT1-1 | wild-type |  |
| C1 | 2008.6.9 | 242499 | 4.9.11 | MAT1-1 | wild-type |  |
| C2 | 2008.6.9 | 242500 | 4.9.11 | MAT1-1 | wild-type |  |
| P1 | 2008.6.9 | 242568 | 4.9.11 | MAT1-1 | wild-type |  |
| P2 | 2008.6.9 | 242569 | 4.9.11 | MAT1-1 | wild-type |  |
| A1 | 2008.6.9 | 242495 | 4.9.11 | MAT1-1 | wild-type |  |
| A3 | 2008.6.9 | 242497 | 4.9.11 | MAT1-1 | wild-type |  |
| M3-6 | 2008 | 726791 | 4.11 | MAT1-2 | wild-type |  |
| M4-2 | 2008 | 726792 | 4.11 | MAT1-2 | wild-type |  |
| MC8 | 2008 | 726793 | 2 | MAT1-2 | wild-type |  |
| T1 | 2008 | 726794 | 0 | MAT1-1 | wild-type |  |
| KO-1 | 2008.6.3. | 242562 | 4 | MAT1-2 | wild-type |  |
| KO-3 | 2008.6.3. | 726795 | 4.11 | MAT1-2 | wild-type |  |
| KO-4 | 2008.6.3. | 726796 | 4.11 | MAT1-2 | wild-type |  |
| KO-5 | 2008.6.3. | 726797 | 4.11 | MAT1-2 | wild-type |  |
| GF922 | 2007.6. | 242547 | 4.11 | MAT1-2 | wild-type |  |
| GF925 | 2007.6. | 726765 | 4.11 | MAT1-2 | wild-type |  |
| GF926 | 2007.6. | 726766 | 4.11 | MAT1-2 | wild-type |  |
| GF928 | 2007.6. | 726767 | 4 | MAT1-2 | wild-type |  |
| GF929 | 2007.6. | 726768 | 4.11 | MAT1-2 | wild-type |  |
| GF931 | 2007.6. | 726769 | 4 | MAT1-2 | wild-type |  |
| GF933 | 2007.10. | 726770 | 4 | MAT1-2 | wild-type |  |
| GF937 | 2007.10. | 726771 | 4 | MAT1-2 | wild-type |  |
| GF939 | 2007.10. | 726772 | 4 | MAT1-2 | wild-type |  |
| GF941 | 2007.10. | 726773 | 4.11 | MAT1-2 | wild-type |  |
| CF312 | 2008.6.23 | 726774 | 4.9 | MAT1-1 | wild-type |  |
| CF313 | 2008.6.27 | 726775 | 0 | MAT1-2 | wild-type |  |
| CF314 | 2008.6.27 | 726776 | 0 | MAT1-2 | wild-type |  |
| CF315 | 2008.6.27 | 726777 | 4.11 | MAT1-2 | wild-type |  |
| CF316 | 2008.6.27 | 726778 | 4 | MAT1-1 | wild-type |  |
| CF317 | 2008.6.27 | 726779 | 4 | MAT1-1 | wild-type |  |
| CF318 | 2008.6.30 | 242533 | 4.9.11 | MAT1-1 | wild-type |  |
| CF320 | 2008.7.1 | 726780 | 0 | MAT1-1 | wild-type |  |
| CF322 | 2008.6.27 | 726781 | 9 | MAT1-1 | wild-type |  |
| CF324 | 2008.6.27 | 726782 | 9 | MAT1-1 | wild-type |  |
| CF328 | 2008.6.25. | 242537 | 0 | MAT1-2 | wild-type |  |
| CF330 | 2008.6.4. | 242538 | 4 | MAT1-2 | wild-type |  |
| CF331 | 2008.6.4. | 726798 | 4 | MAT1-2 | c.-23insT |  |
| CF332 | 2008.6.4. | 726799 | 4 | MAT1-2 | wild-type |  |
| CF333 | 2008.6.4. | 242539 | 4.11 | MAT1-2 | wild-type |  |
| CF334 | 2008 | 242540 | 4.11 | MAT1-1 | wild-type |  |
| CF335 | 2008 | 726800 | 4.11 | MAT1-2 | wild-type |  |
| sy1 | 2005 | 242574 | 4.11 | MAT1-1 | wild-type |  |
| sy2 | 2005 | 242575 | 0 | MAT1-1 | wild-type |  |
| it1 | 2005 | 242561 | 4 | MAT1-1 | wild-type |  |
| H-7 | 2008.6.25 | 242551 | 2.9 | MAT1-2 | wild-type |  |
| H-29 | 2008.6.25 | 242554 | 2.9 | MAT1-2 | wild-type |  |
| H-41 | 2008.7.17 | 242555 | 2.9 | MAT1-2 | wild-type |  |
| 334 | 2007 | 726801 | 4.9 | MAT1-1 | wild-type |  |
| 337 | 2007 | 726715 | 4.9 | MAT1-1 | wild-type |  |
| 341 | 2007 | 726718 | 4.9 | MAT1-1 | wild-type |  |
| 343 | 2007 | 726720 | 4.9 | MAT1-1 | wild-type |  |
| Chi2 | 2007 | 726733 | 4.9.11 | MAT1-1 | wild-type |  |
| C3 | 2008.06.09 | 242501 | 4.9.11 | MAT1-2 | wild-type |  |
| MC3 | 2008 | 726802 | 4.11 | MAT1-2 | wild-type |  |
| 344 | 2008 | 726784 | 4.11 | MAT1-2 | wild-type |  |
| CF310 | 2008 | 726803 | 2 | MAT1-1 | wild-type |  |
| CF311 | 2008 | - | 0 | MAT1-1 | wild-type |  |
| CF304 | 2008 | 242530 | 4.11 | MAT1-1 | wild-type |  |
| Nango1 | 2007 | 726737 | 4.9.11 | MAT1-1 | wild-type |  |
| 11-1 | 2003 | 726649 | 2.4 | MAT1-2 | wild-type |  |
| CF229 | 1998 | 726675 | 0 | MAT1-1 | wild-type |  |
| GF932 | 2007 | 726804 | 4.11 | MAT1-2 | wild-type |  |
| GF936 | 2007 | - | 2 | MAT1-1 | wild-type |  |
| GF938 | 2007 | - | 0 | MAT1-2 | wild-type |  |
| GF940 | 2007 | - | 2 | MAT1-1 | wild-type |  |
| GF942 | 2007 | - | 0 | MAT1-1 | wild-type |  |
| GF1061 | 2009 | - | 2.9 | MAT1-2 | wild-type |  |
| GF1063 | 2009 | - | 2.9 | MAT1-1 | wild-type |  |
| Pf835 | 2010 | - | 2.9 | MAT1-2 | wild-type |  |
| Pf853 | 2010 | - | 2.9 | MAT1-2 | wild-type |  |
| Pf862 | 2010 | - | 2.9 | MAT1-2 | wild-type |  |
| Pf870 | 2010 | - | 2.9 | MAT1-2 | wild-type |  |
| Pf896 | 2010 | - | 2.9 | MAT1-2 | wild-type |  |
| Pf778 | 2010 | - | 2.9 | MAT1-2 | wild-type |  |
| Pf779 | 2010 | - | 2.9 | MAT1-2 | wild-type |  |
| Pf780 | 2010 | - | 2.9 | MAT1-2 | wild-type |  |
| Pf781 | 2010 | - | 2.9 | MAT1-2 | wild-type |  |
| Pf782 | 2010 | - | 2.9 | MAT1-2 | wild-type |  |
| Pf783 | 2010 | - | 2.9 | MAT1-2 | wild-type |  |
| Pf784 | 2010 | - | 2.9 | MAT1-2 | wild-type |  |
| Pf786 | 2010 | - | 2.9 | MAT1-2 | wild-type |  |
| Pf667 | 2010 | - | 4 | MAT1-1 | wild-type |  |
| Pf668 | 2010 | - | 4.11 | MAT1-1 | wild-type |  |
| Pf669 | 2010 | - | 2.4 | MAT1-2 | wild-type |  |
| Pf671 | 2010 | - | 4 | MAT1-2 | wild-type |  |
| Pf674 | 2010 | - | 2 | MAT1-2 | wild-type |  |
| Pf676 | 2010 | - | 2 | MAT1-2 | wild-type |  |
| Pf697 | 2010 | - | 2 | MAT1-2 | wild-type |  |
| Pf698 | 2010 | - | 2 | MAT1-1 | wild-type |  |
| Pf701 | 2010 | - | 2 | MAT1-2 | wild-type |  |
| Pf702 | 2010 | - | 2 | MAT1-1 | wild-type |  |
| Pf730 | 2010 | - | 2.9 | MAT1-2 | wild-type |  |
| Pf731 | 2010 | - | 2.9 | MAT1-1 | wild-type |  |
| Pf732 | 2010 | - | 2.9 | MAT1-1 | wild-type |  |
| Pf733 | 2010 | - | 2.9 | MAT1-1 | wild-type |  |
| Pf738 | 2010 | - | 2.9 | MAT1-1 | wild-type |  |
| Pf739 | 2010 | - | 2.9 | MAT1-1 | wild-type |  |
| Pf740 | 2010 | - | 2.9 | MAT1-1 | wild-type |  |
| Pf741 | 2010 | - | 2.9 | MAT1-1 | wild-type |  |
| Pf743 | 2010 | - | 2.9 | MAT1-1 | wild-type |  |
| GF1546 | - | - | 2.9 | MAT1-2 | wild-type |  |
| GF1547 | - | - | 2.9 | MAT1-2 | wild-type |  |
| GF1548 | - | - | 2.9 | MAT1-2 | wild-type |  |
| GF1549 | - | - | 2.9 | MAT1-2 | wild-type |  |
| GF1550 | - | - | 2.9 | MAT1-2 | wild-type |  |
| GF1551 | - | - | 2.9 | MAT1-2 | wild-type |  |
| GF1552 | - | - | 2.9 | MAT1-2 | wild-type |  |
| GF1553 | - | - | 2.9 | MAT1-2 | wild-type |  |
| Pf2079 | - | - | 2.9 | MAT1-2 | wild-type |  |
| ^a^ The *Cf- ECP20-2* genes were detected in all strains, and all coding sequences were identical to that of the wild-type reference strains WU0; only strain CF331 have a thymine insertion 23 bp before the start codon. | | | | | |  |
|  |  |  |  |  |  |  |


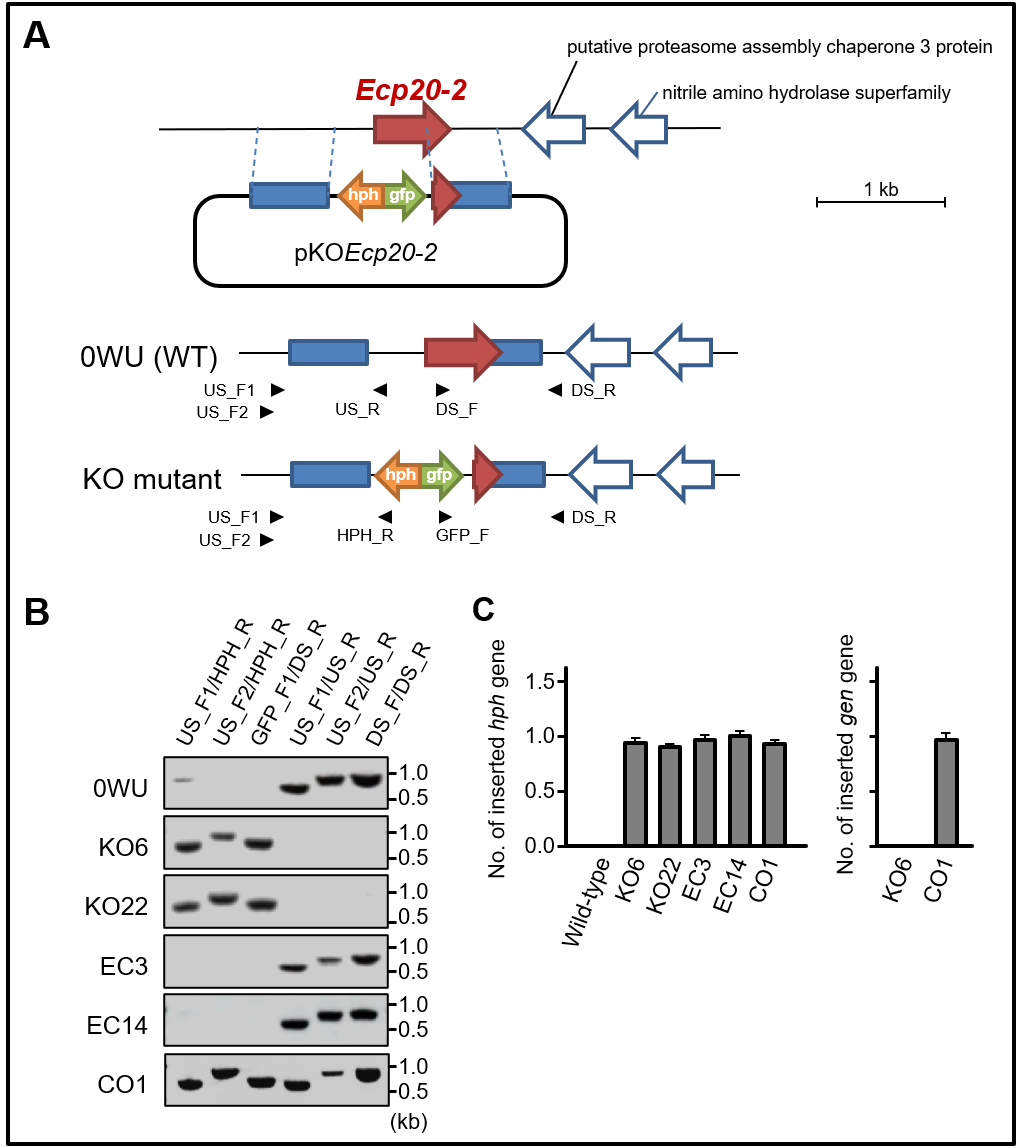


**Figure S1.** Deletion cassette, PCR-cloning, and evaluation of copy numbers in transformants. (A) Representation of the cfgbp locus in the wild-type and knocked-out (KO) mutant strains after homologous recombination. The locus was replaced by hygromycin resistance (*hph*) and green fluorescence protein (*gfp*) genes.

(B) Targeted gene deletion and cfgbp complementation were confirmed by PCR using oligonucleotides shown in panel A. Strains KO6 and KO22, deletion mutants; EC3 and EC14,ectopic transformants; CO1, strain KO6 complemented with  *Ecp20-2* gene.

(C) Single insertion event of the transformation vector was confirmed by quantitative real-time PCR using genomic DNA of each transfomant. The *hph* and genetcine resistance (*gen*) genes were used as a measure for the number of insertion events, together with *C. fulvum* actin genes used for normalization and as a single copy reference gene, respectively, according to the 2^–∆Ct^ method. Error bars represent the standard deviation of three technical replicates.


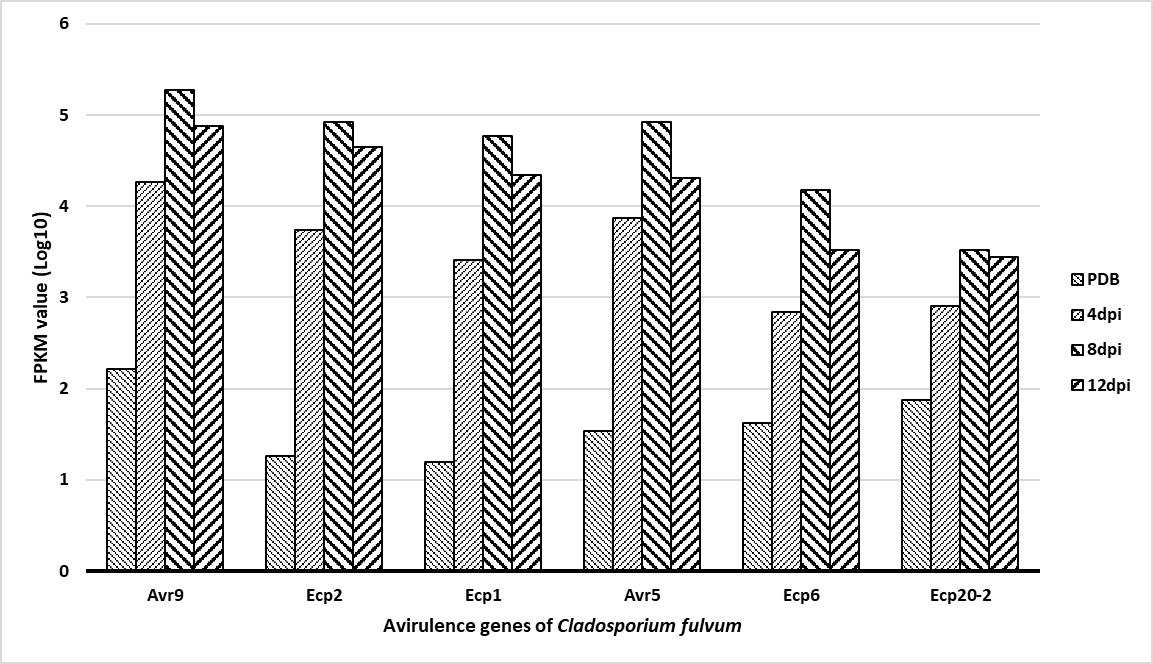


**Figure S2.** FPKM values of *Ecp20-2* and five other known genes of *Cladosporium fulvum.*


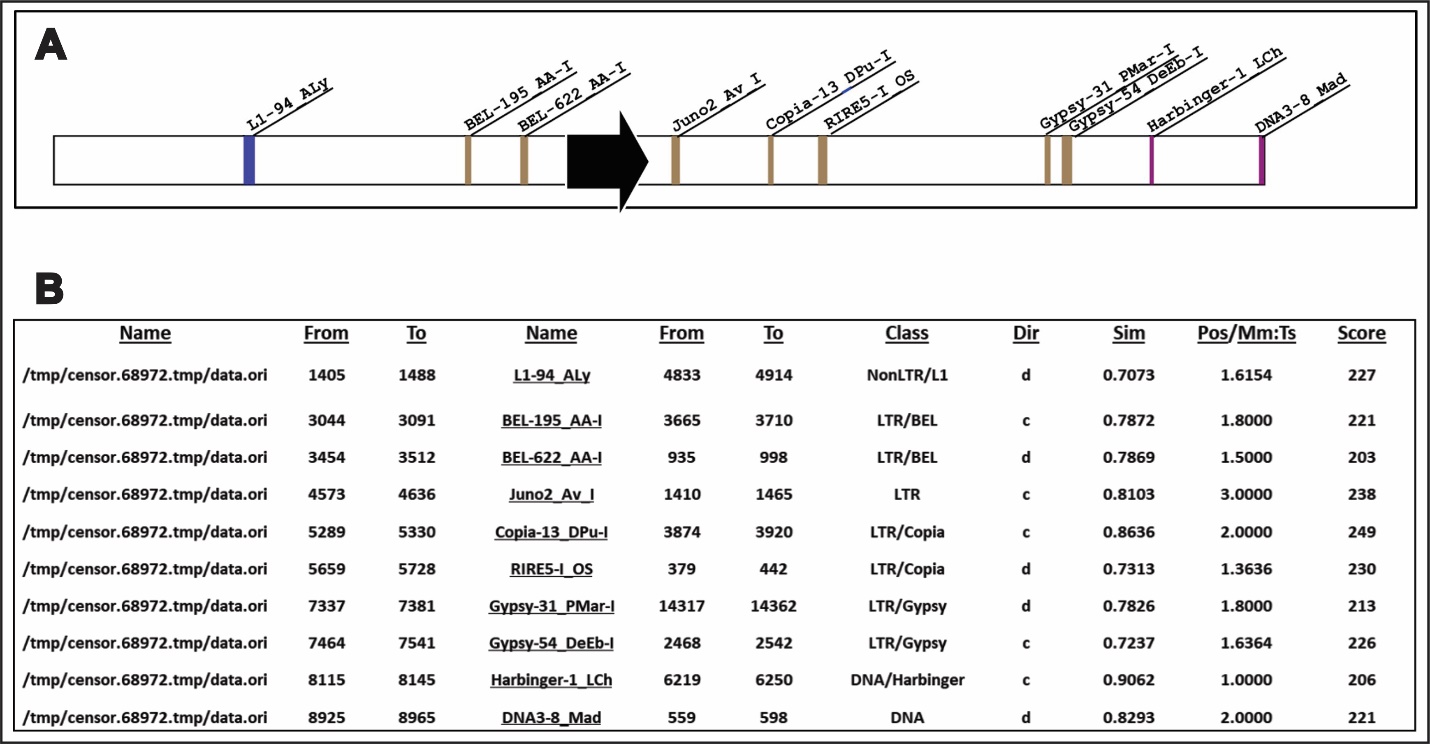


**Figure S3.** Repetitive elements predicted surrounding *Ecp20-2* gene


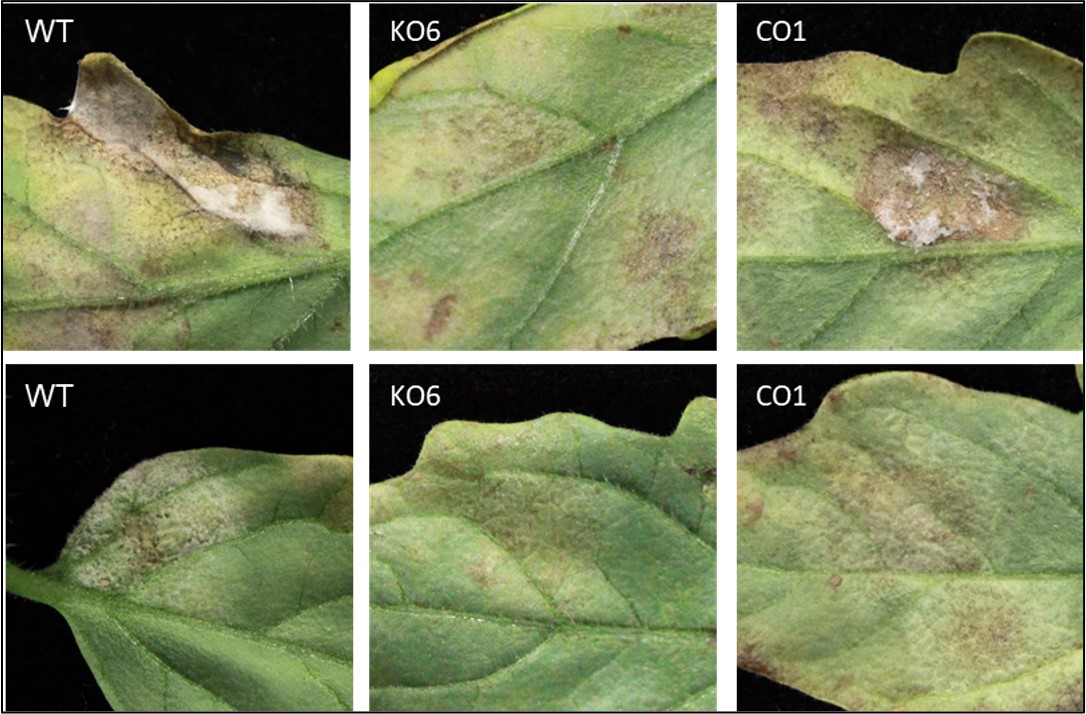


**Fig S4.** Virulence of wild-type strain, *∆ecp20-2* mutant strain KO6, and *ECP20-2* gene-complemented strain CO1 on susceptible tomato plants at 21 days post-inoculation.
